# Supplementary material for: High Numbers of CD163-Positive Macrophages in the Fibrotic Region of Exuberant Granulation Tissue in Horses
Source: Animals (Basel). 2021 Sep 18;11(9):2728. doi: 10.3390/ani11092728 (PMC8464979; doi:10.3390/ani11092728)
Supplement: Supplementary file 1 [file animals-11-02728-s001.zip › animals-1288089-supplementary.pdf]

# Supplementary to

## High numbers of CD163-positive macrophages in the fibrotic region of exuberant granulation tissue in horses.

**Charis Du Cheyne [1], Ann Martens [2] and Ward De Spiegelaere [1,*]**

[1] Department of Morphology, Ghent University, 9820 Merelbeke, Belgium
[2] Department of Surgery and Anaesthesiology of Domestic Animals, Ghent University, 9820 Merelbeke, Belgium
* Correspondence: ward.despiegelaere@ugent.be

This supplementary information includes 5 figures

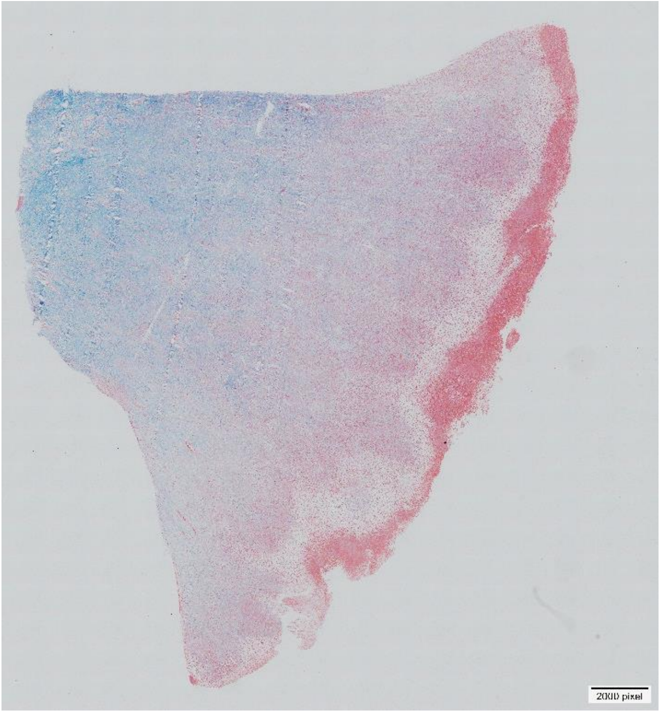


**Figure S1**. Example of a Masson Trichrome staining of EGT. Collagen is stained in blue, showing increasing collagen content in the deeper fibrotic region (left) and limited to no collagen at the level of the wound surface.

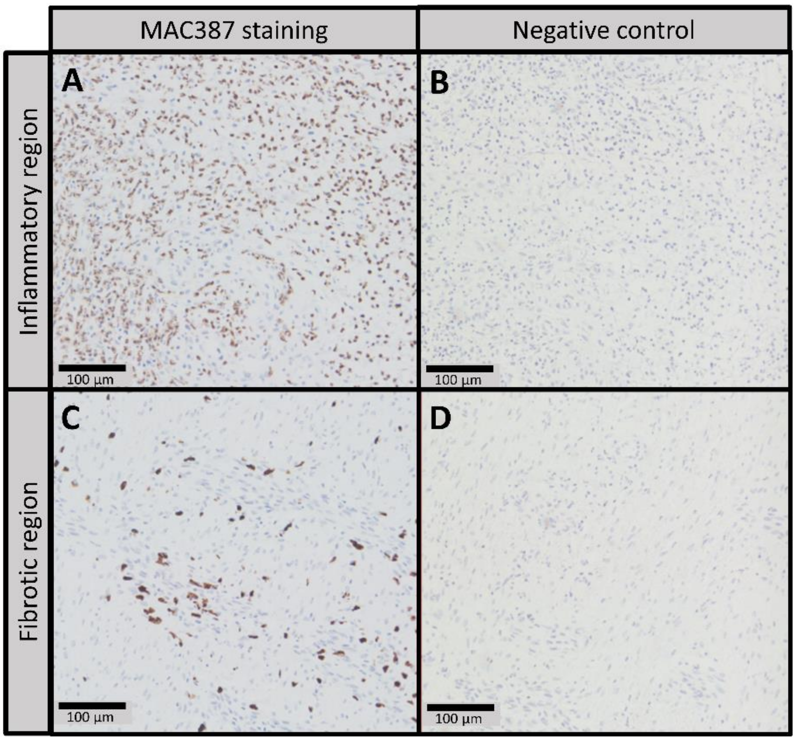


**Figure S2**. Representative pictures of staining versus negative control. A: Inflammatory region of EGT stained for MAC387. B: No primary antibody control of the corresponding region in picture A. C: Fibrotic region of EGT stained for MAC387. D: No primary antibody control of the corresponding region in picture C.

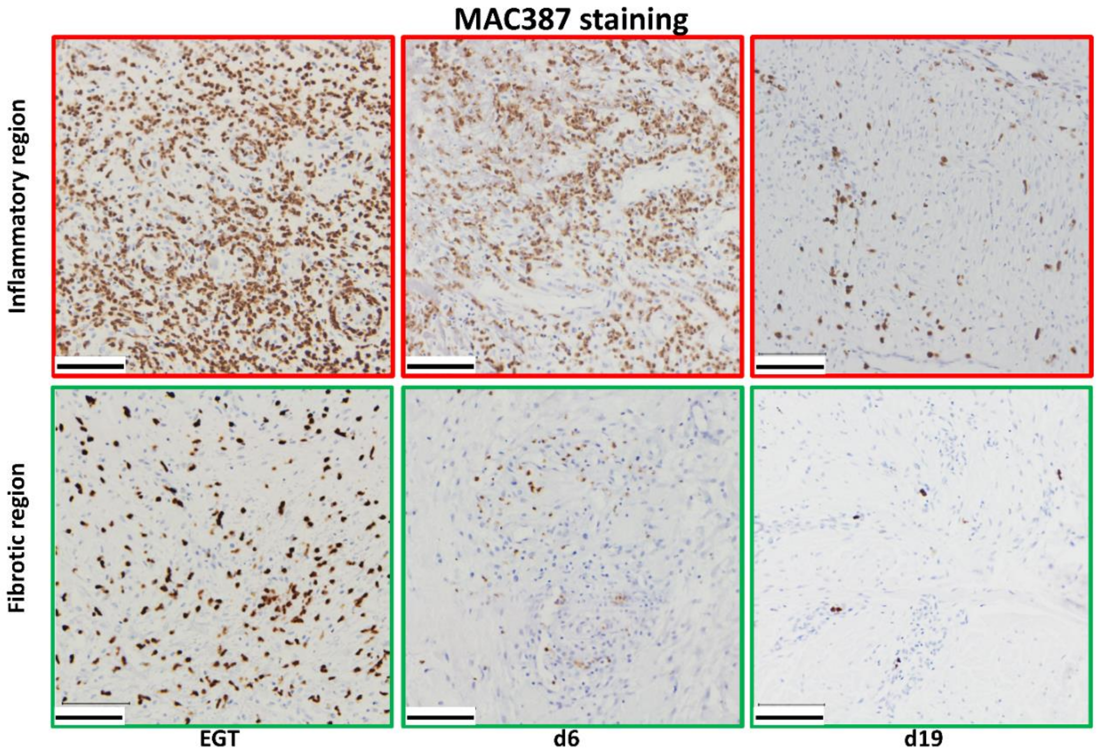


**Figure S3**. The original/raw figures for the MAC387 staining used as input for the analysis in QuPath corresponding to the processed images in Figure 4A.

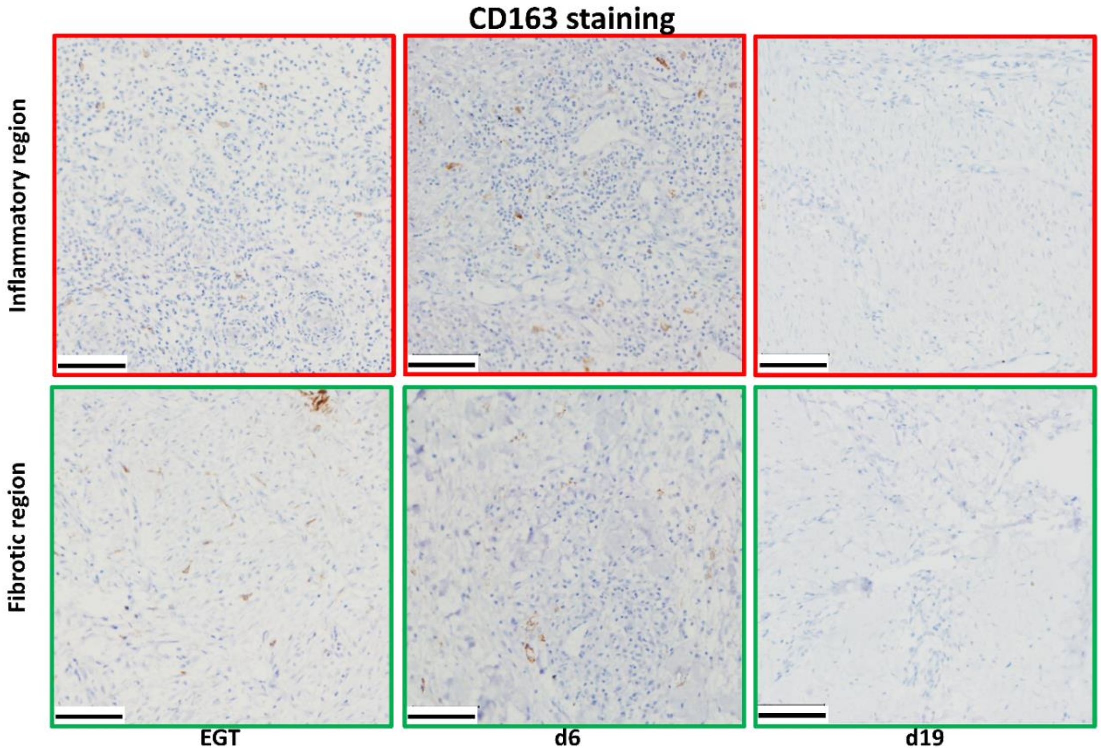


**Figure S4**. The original/raw figures for the CD163 staining used as input for the analysis in QuPath corresponding to processed images in Figure 5A.

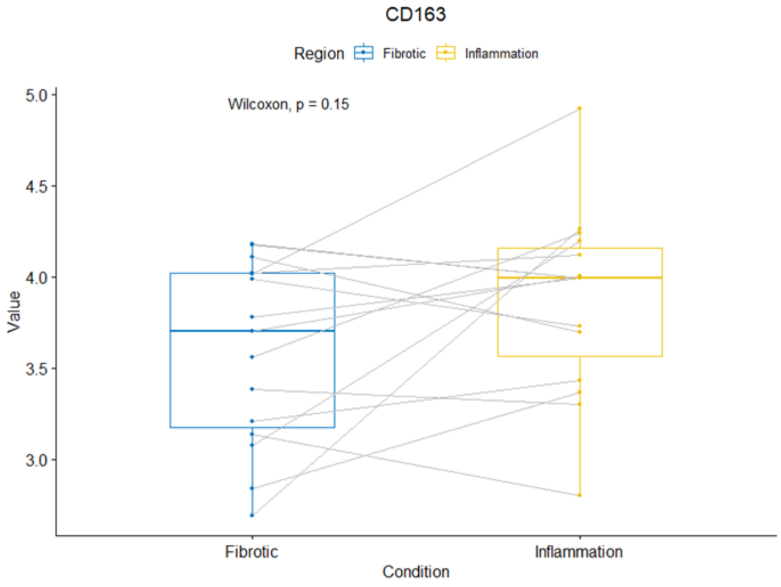


**Figure S5**. Boxplot comparing CD163 staining between fibrotic and inflammatory regions within wounds. No significant difference was found regarding CD163 staining between the pooled fibrotic regions on one hand and the pooled inflammation regions on the other hand with a Wilcoxon signed-rank test. Pooled: all samples for the three groups i.e. EGT, experimental wound 6 days post injury and experimental wound 19 days post injury were taken together.
